# Human attitudes towards herpetofauna: The influence of folklore and negative values on the conservation of amphibians and reptiles in Portugal

**DOI:** 10.1186/1746-4269-8-8

**Published:** 2012-02-08

**Authors:** Luis MP Ceríaco

**Affiliations:** 1 Centro de Estudos de História e Filosofia da Ciência (CEHFCi), Universidade de Évora, Palácio do Vimioso, Largo Marquês de Marialva, n°8, 7000-554, Évora, Portugal

## Abstract

**Background:**

Human values and folklore of wildlife strongly influence the effectiveness of conservation efforts. These values and folklore may also vary with certain demographic characteristics such as gender, age, or education. Reptiles and amphibians are among the least appreciated of vertebrates and are victims of many negative values and wrong ideas resulting from the direct interpretation of folklore. We try to demonstrate how these values and folklore can affect the way people relate to them and also the possible conservation impacts on these animals.

**Methods:**

A questionnaire survey distributed to 514 people in the district of Évora, Portugal, was used to obtain data regarding the hypothesis that the existence of wrong ideas and negative values contributes to the phenomenon of human-associated persecution of these animals. A structural equation model was specified in order to confirm the hypothesis about the possible relationships between the presence of perceptions and negative values about amphibians and reptiles and persecution and anti-conservation attitudes. Sociodemographic variables were also added.

**Results:**

The results of the model suggest that the presence of folklore and negative values clearly predicts persecution and anti-conservation attitudes towards amphibians and reptiles. Also, the existence of folklore varies sociodemographically, but negative values concerning these animals are widespread in the population.

**Conclusions:**

With the use of structural equation models, this work is a contribution to the study of how certain ideas and values can directly influence human attitudes towards herpetofauna and how they can be a serious conservation issue.

## Background

Not all animal species, whether endangered or not, are fortunate enough to be appreciated by humans. While it is true that aesthetic reasons are not (or should not be) scientifically accepted when carrying out conservation measures, the fact remains that aesthetics greatly influences the support given by the public and various decision-making bodies to the preservation of many species [1]. It is easier to justify the preservation of more aesthetically pleasant species than less appreciated species [2]. Considering this, species like the giant panda (*Ailuropoda melanoleuca*) and dolphins are often used as symbols by famous organizations or environmental protection agencies and are called "flagship species". They are ambassadors for conservation and their protection contributes to the preservation of other organisms in their ecosystems [3,4]. Human preferences among different types of organisms have influenced the provision of conservation resources toward large charismatic species [5] and what are largely considered by the public to be more attractive vertebrate groups [6]. Birds, mammals, and fishes may have been more privileged and protected because they are more socially accepted than reptiles, amphibians, and invertebrates [7]; however, there are exceptions [8]. For example, bats are also mammals, but they are regarded as similar to reptiles or invertebrates. The Iberian wolf (*Canis lupus signatus*) is another exception to this rule and illustrates the factors affecting wildlife conservation. In Portugal the wolf's image (e.g., as a bloodthirsty, demonic, man-eating animal) has been influenced by negative values, folklore, and mythologies and it has been perceived as a threat to regional pastoralist economies [9]. Fear and competition for food create a conflict between the wolf and man, leading to persecution and deliberate extermination of wolves [9]. The perception that a particular animal is dangerous and aggressive to humans, like the Iberian wolf in Portugal, has led to other similar situations for other large carnivores all over the world, as documented in other studies [10-20].

Although reptiles and amphibians are not responsible for major economic losses and most are harmless, they are feared and persecuted [21,22]. In fact, many reptiles are quite useful for human beings, not only as sources of food [22], medicines [23], and raw materials [21], but also in terms of ecological equilibrium. Despite this usefulness to human beings, many animals are seen as dangerous and are persecuted. For instance, despite its ecological importance and role in preventing mosquito plagues, the gecko is seen as a poisonous and evil animal and is therefore persecuted in Portugal [24]. Knight [8] showed that most people disdain creatures that represent little threat to humans. These fears are often irrational and might be connected to animal phobias [12], cultural issues [12,25], and emotional reactions [8]. Aspects of human evolution might also have led to fears of these animals. Sagan [26] suggests that the human fear of reptiles could be a result of the ancient conditions in which the first mammals evolved. In addition to that, he suggests that these fears may even be an evolutionary heritage. The high prevalence of fear of snakes and other animals among humans and other primates suggests that this fear is the result of an ancient evolutionary history, and genetic variability may explain why not all individuals harbor these phobias [27,28].

Morris and Morris [29] reported that in Britain 27% of the children interviewed stated that snakes were the least liked animal and that 24% of people said that snakes were the animals whose conservation status they cared about least. Agras et al. [30] reported that in a U.S. study, fear of snakes was the most intense compared with other animal phobias in the country, and this fear was prevalent in 38% of women and 12% of men interviewed. Fear of certain animals constitutes a great number of phobias, such as fear of spiders (arachnophobia), insects (insectophobia), rats (musophobia), and snakes (ophidiophobia), with snakes being at the top of the list globally [31]. Phobias associated with snakes and spiders are the most common phobias in Western societies and may result in part from genetic predisposition associated with the risk experienced by humans during their evolution, resulting in a process known as biophobia [32]. These types of feelings towards animals are what Kellert [14,25,33] defines as negative values in his typology of attitudes towards animals.

The causes of human persecution of animals have various natures [17,19,20,24,34], and the existence of a large number of myths, stories, and misconceptions (some of them resulting from the direct interpretation of local folklore) may be largely responsible for some of this persecution [19,22]. In Portugal, there are a large number of such folklore tales about reptiles and amphibians [22,24,35,36], mostly depicting reptiles and amphibians as evil and dangerous animals. These types of misconceptions are just more ideas to add to the vast list of erroneous ideas and negative values about reptiles and amphibians. The idea of threat or potential harm to humans is one of the main reasons for disliking animals, and the prevalence of perceptions rather than actual bio-ecological characteristics is also the most important reason for the preference for certain types of animals.

All of those folklore, ideas, perceptions, and values are a very important part of the human relation with animals (besides the more "scientific" zoological approaches) and can be considered as a part of the human relation with animals, or "ethnozoology". According to Alves et al. [37] we can define ethnozoology as "the variety of interactions (both past and present) that human cultures maintain with animals" and this type of study "has its roots as deep within the past as the first relationships between humans and other animals". Although dealing with a very vast and important area--all the types of human relations with animals--these studies are still not very common worldwide, except in Brazil [37], where many studies have already been done. In this regard, as a sub-part of ethnozoology, ethnoherpetological studies are even less common worldwide. Ethnoherpetology can be defined as the study of people's relations with and knowledge about reptiles and amphibians. Worldwide there are few studies on the topic, and existing ones are mainly concentrated in Africa [38-40], south America [41-45], and Asia [46-49]. In Europe these types of studies are very rare [50-52] and in Portugal, apart from some anecdotal references in some herpetological publications or in old general ethnographic studies, there are also few studies on the topic [24,35,36]. Studies presenting situations in which this type of knowledge has a negative impact on conservation are few, and almost none have ever established a clear link between the presence of folklore, negative values, and preferences and persecution and anti-conservation attitudes towards reptiles.

It is therefore extremely important to understand how common these tales, folklore, misperceptions, and negative values are in societies and how these factors can contribute to the persecution of reptiles and amphibians and affect their conservation. Although the theme is quite common and is reported as anecdotal information in the literature on herpetology, conservation biology, and ethnozoology [3,4,21,22,35], few studies have been dedicated to understanding how the presence of these topics may or may not influence peoples' attitudes towards reptiles and amphibians. This work is intended to be an early contribution to clarify the situation. Thus, the general objectives of this study were to analyze human values and folklore about herpetofauna and their possible relationship with the persecution which this fauna suffers.

## Methods

### Study area and participants

This study was conducted in the district of Évora, southern Portugal, from January to July 2009. The surveys were conducted in two urban areas, Évora and Montemor-o-Novo, and six rural areas, Valverde, Evoramonte, Mora, Borba, Mourão, and Vila-Viçosa. Participants were randomly selected in public places such as public squares, waiting rooms, schools, shops, cafes, and homes in the various locations of the study. The aim was to obtain a representative sample of the population. The minimum age of survey respondents was 14 years and the maximum was 81 years. A total of 514 persons participated in this investigation, comprising 261 males and 253 females, 283 from urban areas and 231 from rural areas. Informed consent was given by those interviewed.

### Species focused on in the study

We created two major study groups, amphibians and reptiles, following the Portuguese folk taxonomy for amphibians and reptiles, and these two were then divided into nine minor groups, each representing a particular type of animal among the herpetofauna of the region, equivalent in most cases to the family taxon (Table 1). Therefore the reptile category comprised six groups, namely turtles, geckos, small lizards, big lizards, snakes, and vipers, representing 16 species that occur in the study region [53] (see Table 1), and the amphibians category comprised three groups, namely salamanders & newts, toads, and frogs, representing 14 species that occur in the study region [53] (see Table 1).

**Table 1:** Groups presented in the study and the species represented by each group

Group		Species represented and IUCN Conservation status in Portugal
Amphibians	Salamanders and newts	*Salamandra salamandra *(LC)
		
		*Pleurodeles waltl *(LC)
		
		*Triturus boscai *(LC)
		
		*Triturus marmoratus *(LC)
	
	Frogs	*Pelophylax perezi *(LC)
		
		*Discoglossus galganoi *(NT)
		
		*Hyla arborea *(LC)
		
		*Hyla meridionalis *(LC)
	
	Toads	*Pelobates cultripes *(LC)
		
		*Pelodytes punctatus *(NE)
		
		*Alytes cisternasii *(LC)
		
		*Alytes obstetricans *(LC)
		
		*Bufo calamita *(LC)
		
		*Bufo bufo *(LC)

Reptiles	Turtles	*Mauremys leprosa *(LC)
		
		*Emys orbicularis *(EN)
	
	Geckos	*Hemidactylus turcicus *(VU)
		
		*Tarentola mauritanica *(LC)
	
	Small lizards	*Podarcis hispanica *(LC)
		
		*Psammodromus hispanicus *(NT)
		
		*Psammodromus algirus *(LC)
	
	Big lizards	*Timon lepidus *(LC)
	
	Snakes	*Coluber hippocrepis *(LC)
		
		*Coronella girondica *(LC)
		
		*Elaphe scalaris *(LC)
		
		*Macroprotodon cucullatus *(LC)
		
		*Natrix maura *(LC)
		
		*Natrix natrix *(LC)
		
		*Malpolon monspessulanus *(LC)
	
	Vipers	*Vipera latastei *(VU)

### Procedure

Participants were randomly selected in public places, informed about the methodology and objectives of the study, and, after obtaining their informed consent to participate, the data were collected and respondents were assured that their identities would remain confidential. All of the interview and questionnaire followed a pre specified protocol to assure that no differences would exist between interviews. The questionnaire was accompanied by a pamphlet with photos representing the various animals in the study. All the surveys were done during the day between 10.00 and 17.00 hours, and were done presentially. After the data were collected, all issues concerning the herpetofauna were then clarified in a brief leaflet about environmental awareness to address some doubts and misconceptions shown by the respondents. The whole process of data collection and environmental information took between 20 to 30 minutes.

### Questionnaire construction and measures

Three different scales were built, each representing a different latent variable or construct, and turned into a questionnaire. The latent variables are hypothetical constructs which are not measured directly but estimated from a set of indicator items [54,55] that can be directly observed and measured. As for the development of the three different constructs, beliefs and generalized ideas about reptiles and amphibians in the entire Portuguese population were collected. This collection was carried out based on conversations about the animals with different people, namely university ecologists, biologists, and sociologists, and also by collecting literature in several books and documents. The scale for measuring the construct "folklore" was generated on the basis of general ideas and features that local people associate with wildlife, especially reptiles and amphibians, in Portugal. It was initially composed of 11 items, of which three were formulated in a positive way (e.g. "They are important to the ecosystem" or "It's completely harmless"), while nine were negatively worded (e.g. "When one of these animals sees a human, the animal usually attacks" or "They are dangerous to humans"). Nine items were statements regarding the bio-ecological characteristics of animals (e.g. "It's poisonous" or "It's important to the ecosystem"), while the other three referred to the perception of their behaviors. Participants were invited to express their views on a range of 10 values dedicated to these constructs, ranging from zero ("I totally disagree") to 10 ("I totally agree"). Although that folklore related to amphibians and reptiles is much more complex than the present items, these statements are simplified versions of the local stories and ideas, presenting the basis of those. The "negative values" construct consisted of 11 statements revealing negative feelings and/or fears towards different animals. Examples of items for this scale are "I like the animal" or "The animal gives me nightmares." Participants were invited to express their views according to a range of 10 values, ranging from zero "Does not apply to me" to 10 "Fully applies to me." A 10-value scale was created to measure the "persecution/anti-conservation" construct. This scale contains items that refer to legislation (for example: "I agree that these animals are protected by law") and the relationship that people have with animals (for example: "I tend to kill them when I encounter them" or "If there was a population of these animals in my yard, I would take measures to eradicate it." Participants were again asked to express their views on a 10-value scale ranging from zero "Does not apply to me" to 10 "Totally applies to me." Reliability analyses for the scales used were conducted by considering Cronbach's alpha as an indicator of internal consistency [54]. One-way ANOVA tests (p < .05) were used to explore how the perceptions and negative values (dependent variables) varied with the sociodemographic factors of location of residence, gender, education, and age.

### Structural equation modeling

The use of structural equation models (SEMs) is common in investigations in several fields including conservation biology [56], environmental psychology [8,57], and human dimensions of wildlife [58]. We specified an SEM using AMOS (SPSS Inc., Chicago, Illinois, USA) in order to confirm the hypothesized relationships of conservation with the rest of the assessed factors. SEM contains two identifiable models: the measurement model and the structural model. The first one is basically a confirmatory factor analysis in which the relations between every factor and its supposedly observed variables are specified [59] and the validity of these relations is tested. High and significant lambdas (i.e., factor loadings) are indicators of convergent construct validity for the assessed factors. In turn, the structural model contains the relations between factors as well as the relations between manifest variables and latent factors. In addition, goodness of fit indicators (chi-squared, NNFI, and CFI practical goodness of fit indexes, RMSEA, etc.) reveal whether or not the data support the adequacy of the hypothesized factor structure and the pattern of presumed interrelations between factors [54,59,60]. The overall fit of the partial mediation model was assessed using five indicators (*X*^2^, *X*^2^/df, NNFI, CFI, and RMSEA). Marsh and Hocevar [61] suggest that the chi-square should be evaluated in relation to the model's degrees of freedom; an *X*^2^/df ratio of 2:1 to 5:1 indicates an acceptable fit [60]. Values higher than 0.90 indicate acceptable fit of the CFI and NNFI indicators [60]. Values for the RMSEA should be less than 0.08 for acceptable fit. In this study, three factors were pre-specified within the first SEM to be tested. They were perceptions, negative values, and persecution/anti-conservation (endogenous factors). Age, location, education, and gender were added into the model as demographic covariates (independent factors). Folklore, negative values, and the demographic indicators were specified as predictors of persecution. Covariances between the endogenous factors and the rest of the independent factors were estimated. Then we tested two SEMs: one for reptiles and another for amphibians.

## Results

### Invariable statistics

The invariable statistics (Table 2) demonstrate that the influence of folklore (mean = 4.6) and negative values (mean = 4.6) is higher for reptiles (mean = 3.8) than for amphibians (mean = 3.4). Persecution is higher for reptiles (mean = 3.8) than for amphibians (mean = 2.4). In all cases the scales used showed good reliability coefficients (Cronbach's alphas), with alpha equal to 62% or higher (Table 2).

**Table 2:** Univariate statistics and reliability (Cronbach's alpha) of scales used

Scale/items	Reptiles					Amphibians				
	Mean	SD	**Min**.	**Max**.	Alpha	Mean	SD	**Min**.	**Max**.	Alpha
***Folklore***	*4.56*	*1.82*	*0*	*10*	*0.67*	*3.84*	*.73*	*0*	*10*	*0.62*
are dangerous animals	3.85	1.70	0	10		2.21	2.34	0	10	
are useful animals^a^	7.25	2.91	0	10		6.71	3.26	0	10	
are poisonous	3.96	2.36	0	10		2.67	2.88	0	10	
are fatal to human beings	5.10	7.82	0	10		5.12	3.86	0	10	
are completely inoffensive^a^	5.30	2.50	0	10		5.89	3.33	0	10	
usually attack humans	2.71	2.23	0	10		1.22	1.87	0	10	
usually ignore humans^a^	5.88	2.99	0	10		4.54	4.27	0	10	
usually flee from humans^a^	3.85	2.58	0	10		3.43	3.25	0	10	
are important to the ecosystem^a^	3.14	3.82	0	10		2.74	3.37	0	10	
***Negative values***	*4.63*	*2.06*	*0*	*10*	*0.73*	*3.37*	*0.69*	*0*	*10*	*0.71*
I like the animal^a^	7.62	2.30	0	10		7.23	3.32	0	10	
I find the animal ugly	5.53	3.65	0	10		5.11	3.65	0	10	
I don't go near places where the animal is	4.78	3.29	0	10		3.42	3.45	0	10	
The way the animal moves gives me the creeps	3.63	3.82	0	10		2.36	3.12	0	10	
I don't like the noises that the animal makes	2.14	3.05	0	10		2.00	3.20	0	10	
I fear the animal	4.39	7.88	0	10		2.14	2.97	0	10	
I'm sick of the animal	4.43	3.21	0	10		4.09	4.30	0	10	
The animal gives me nightmares	.98	2.16	0	10		.61	1.87	0	10	
I don't mind if the animal lives in my house/property^a^	7.27	3.21	0	10		-	-	-	-	
I think that the presence of the animal gives value to the surrounding environment^a^	5.54	3.72	0	10		-	-	-	-	
***Persecution/anti-conservation***	*3.79*	*1.13*	*0*	*10*	*0.67*	*2.41*	*.79*	*0*	*10*	*0.67*
When I find one of these animals, I usually kill it or ask someone to kill it	2.98	3.29	0	10		1.91	3.01	0	10	
If there was a population of these animals in my property I would take measures to eliminate it	4.69	3.69	0	10		3.32	3.66	0	10	
I think there should be a greater concern for the preservation of these animals^a^	4.84	3.44	0	10		-	-	-	-	
I think that these animals should be exterminated	2.66	3.47	0	10		2.01	3.03	0	10	

Both scales ranged from 0 to 10.

^a ^Reversed

The presence of such folklore about reptiles varied between locations of residence (*p *< .05), with rural people having it more present than urban people, between educational levels (*p *< .001), with less educated people having also it more present than more educated ones, and between ages (*p *< .001), with younger people more affected that older people (Table 3). For amphibians, folklore varied significantly only between ages (*p *= .001), with older people having more of this ideas than younger ones, and between educational levels (*p *< .001) with, surprisingly, more highly educated people having presenting more folklore ideas than less educated people (Table 3).

**Table 3:** Folklore by sociodemographic variables

	Sample		Dependent variable: Folklore about reptiles				Dependent variable: Folklore about amphibians			
Demographics	N	%	Mean	SD	F value	p value	Mean	SD	F value	p value
Locale					6.603	.010			1.311	.253
- Urban	283	57	4.21	1.36			4.37	1.38		
- Rural	231	43	5.53	1.42			4.24	1.24		
Gender					.342	.559			.297	.586
- Male	261	51	4.39	1.38			4.28	1.31		
- Female	253	49	4.32	1.40			4.35	1.33		
Education					6.052	.000			5.682	.001
- 4th grade	114	22	4.20	1.47			4.17	1.09		
- 9th grade	199	40	4.69	1.26			4.12	1.36		
- 12th grade	119	24	4.14	1.41			4.56	1.27		
- BSc or Higher	69	14	4.02	1.45			4.71	1.48		
Age					6.955	.000			9.682	.000
- 0-14 years	95	18	4.83	1.36			3.89	1.40		
- 15-24 years	141	27	4.34	1.40			4.33	1.20		
- 25-64 years	162	32	4.08	1.41			4.71	1.53		
- 65 or more years	116	23	4.38	1.29			4.09	.86		

^a ^The scale of wrong perceptions on reptiles and amphibians ranges from 0 (participant did not agree with the statement about wrong perceptions of reptiles and amphibians) to 10 (participant totally agreed with the statement).

Cronbach's alpha for reptiles = 67%

Cronbach's alpha for amphibians = 62%

A negative value reflects the fact that the disdain for reptiles is widespread throughout the population, and no significant differences were found between any of the sociodemographic variables. In the case of amphibians, the only variable that showed significant differences was gender (*p *< .001), with women showing more dislike for amphibians than men (Table 4).

**Table 4:** Negative values by sociodemographic variables

	Sample		Dependent variable: Negative values about reptiles				Dependent variable: Negative values about amphibians			
Demographics	N	%	Mean	SD	F value	p value	Mean	SD	F value	p value
Locale					.241	.624			4.342	.038
- Urban	283	57	4.58	2.33			2.66	1.98		
- Rural	231	43	4.68	1.84			3.01	1.76		
Gender					1.430	.232			35.760	.000
- Male	261	51	4.51	2.27			2.33	1.61		
- Female	253	49	4.74	1.97			3.29	2.02		
Education					1.731	.160			.547	.650
- 4th grade	114	22	4.92	1.83			2.69	1.36		
- 9th grade	199	40	4.50	2.34			2.94	2.07		
- 12th grade	119	24	4.76	1.95			2.76	1.88		
- BSc or Higher	69	14	4.29	2.14			2.74	2.06		
Age					.372	.774			1.672	.172
- 0-14 years	95	18	4.52	2.13			3.15	2.09		
- 15-24 years	141	27	4.52	1.80			2.84	2.03		
- 25-64 years	162	32	4.70	2.19			2.77	2.06		
- 65 or more years	116	23	4.74	2.38			2.57	1.11		

^a ^The scale for negativie values about reptiles and amphibians ranges from 0 (participant said that the statement about negative values about reptiles and amphibians did not apply to him/her) to 10 (participant said the statement totally applied to him/her)

Cronbach's alpha for reptiles = 73%

Cronbach's alpha for amphibians = 71%

### Structural equation model

**Figure 1:**
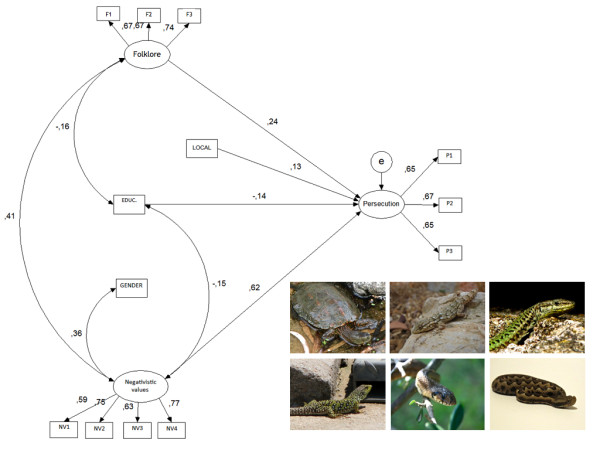
**Test of the model of relations between folklore, negative values, sociodemographic variables, and persecution, for reptiles**. Structural coefficients and factor loadings are standardized. Goodness of fit: *X*^2 ^= 191 (d.f. 60); *p *= .000; NNFI = .90; CFI = .92; RMSEA = .07. Persecution R^2 ^= .84. F1--are dangerous animals; F2--are poisonous; F3--are fatal to human beings; NV1--I find the animal ugly; NV2--the way the animal moves gives me creeps; NV3--I don't go near places where the animal is; NV3--I'm sick of the animal; P1--when I find one of these animals, I usually kill it or ask someone to kill it; P2--I think that these animals should be exterminated; P3--if there was a population of these animals in my property I would take measures to eliminate it.

All of the lambdas were significant (*p *< .05) and the values of covariances between factors were lower than the values of those lambdas, which indicates divergent (discriminant) validity. For reptiles Figure 1 shows that the presence of wrong perceptions and negative values directly influences persecution and anti-conservation attitudes. Folklore had a 0.41 correlation with negative values. Gender was significantly correlated with negative values with a correlation of 0.36. Location of residence was not significantly correlated with any other factor, while education was significantly correlated with negative values and with folklore, with correlations of -0.15 and -0.16 respectively.

Folklore and negative values were positively and significantly linked to persecution, with salient structural coefficients of .24 (*p *< .001) and .62 (*p *< .001) respectively. The sociodemographic characteristics locale and education were significantly linked to persecution: people with lower levels of education were more likely to persecute these animals than those with higher levels of education (*p *= -.14; *p *= .004) and rural people were more likely to do so than urban residents (*p *= .13; p = .01). These four variables explained 84% of the variance of the persecution and anti-conservation attitudes.

**Figure 2:**
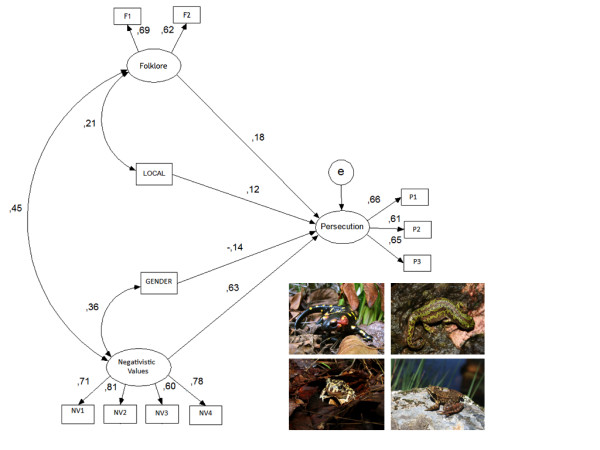
**Test of the model of relations between folklore, negative values, sociodemographic variables, and persecution, for amphibians**. Structural coefficients and factor loadings are standardized. Goodness of fit: *X*^2 ^= 98.3 (d.f. 39); *p *= .000; NNFI = .94; CFI = .96; RMSEA = .05. Persecution R^2 ^= .78. F1--are dangerous animals; F2--are poisonous; NV1- the way the animal moves gives me creeps; NV2--I don't go near places where the animal is; NV3--I'm sick of the animal; NV4--I fear the animal; P1--when I find one of these animals, I usually kill it or ask someone to kill it; P2--I think that these animals should be exterminated; P3--if there was a population of these animals in my property I would take measures to eliminate it.

For amphibians, Figure 2 shows that the presence of wrong perceptions and negative values directly influences persecution and anti-conservation attitudes. The presence of folklore was also related to the presence of negative values; that is, people who have folklore also have negative values towards amphibians. Gender also affects the presence of negative values, with women having more negative values towards these animals than men, and locale of residence also influenced the presence of folklore, with urban people being more likely to have these ideas about amphibians. The sociodemographic characteristics locale and education were significantly linked to persecution with a structural coefficient of .12, while gender had a significant link to persecution, with a salient structural coefficient of .-14, indicating that men are more likely to persecute amphibians than females. Although the *p*-value associated with the statistical indicator chi-squared was found to be significant, the values of NNFI and CFI were higher than .90 and the RMSEA value was .055, indicating that the data supported the specified model [54,59,60]. These four variables explained 78% of the variance of the persecution and anti-conservation attitudes.

## Discussion

What people feel and believe about the environment determines their attitudes towards it [62]. The results suggest that the human persecution and anti-conservation attitudes towards reptiles and amphibians are explained by the presence of folklore and negative values and that sociodemographic variables also affect the presence of these ideas and values. Also, folklore about reptiles and amphibians showed a significant relationship with negative values towards these animals. These results are similar to those of Prokop and Tunnicliffe [63], who found that wrong perceptions about bats and spiders, resulting from the interpretation of common folklore, also showed a significant relationship with negative values. Also, the results of Coursey [5], Czech et al. [6], and Czech and Krausman [7] already indicated a preference for other animals to the detriment of amphibians and reptiles, even anticipating that the support dedicated to their preservation would be less than that provided to other animals [1,2,8,64]. More recently Nolan et al. [65], has also shown that snakes and "wugs", a taxon that include invertebrates like snails, crabs, but also vertebrate as lizards and turtles, were the least appreciated animals when compared to other taxons as mammals, birds or fishes. As the authors state [65], that emotional responses to animals comprise an important dimension in the retention and articulation of ethnobiological information. It can be concluded that these wrong perceptions, resulting from folklore, can clearly influence the attitudes people have towards these animals. However, it must be taken into account that not all of the wrong perceptions about amphibians and reptiles result directly from folklore, such, as it was already referred, evolutionary responses, or lack of information, can lead people to picture these animals as dangerous, lethal or aggressive. Reptiles were more misunderstood than amphibians, and, in fact, amphibians showed lower negative valorization than reptiles. It would be of great interest to better explore this relation between folklore and the values expressed by people, since the first can be one of the reasons for the aversion showed by people towards the herpetofauna. Also, reptiles are more phobia-inducing animals than amphibians, because from even an evolutionary point of view reptiles posed real threats to mammals while amphibians did not [26-28].

These presence of folklore varied significantly with place of residence, age, and level of education, in the case of reptiles, and with age and education in the case of amphibians. Generally, older people had fewer of these folklore related wrong perceptions than younger people, and this can be explained by their higher levels of experience and knowledge. However, as we expected, negative values about these animals are also widespread in the population, having greater values for reptiles than for amphibians.

There is a common belief that aesthetics is an important determinant of public support for species protection, and this is supported by the studies of Stokes [66] and Knight [8]. In our study, we did not examine this aesthetics factor, but we studied negative values. Kellert [25] states that a negative value is indicated when people show feelings of fear, aversion, and dislike for some species of animals. We argue that some part of these negative values could be based on aesthetic arguments, so when exploring the negative values we may also be exploring aesthetics. Also, as Knight [8] states, some of our emotional responses to some animals, such as snakes and spiders, may be guided by not only our fear but also our aesthetic preferences. Our conclusions present one of the first evidences that the presence of folklore and negative values clearly affects the conservation of amphibians and reptiles, and therefore should be taken into account when dealing with conservation problems.

## Conclusions

The persecution triggered by these factors can be a serious factor that threatens populations of amphibians and reptiles, especially reptiles, since they are the most feared and hated, and more species with problematic classifications (Table 1). The problem of direct persecution of herpetofauna is a major threat to the survival of some reptile species, endangered or not, from Europe [67]. The greatest threats to reptiles and amphibians are due to habitat destruction, pollution, climate change, and competition with alien species [68], but it is also known that the complex relationship between humans and these animals, consisting in their direct persecution, capture, and killing, poses a serious and real threat. Cox et al. [68] report that in the Mediterranean basin, the greatest threat to reptiles is the destruction and alteration of habitat, affecting not only endangered species but also species that are not yet threatened, followed by over-exploitation of animals, pollution, and invasive species. In the same report the authors also state that many species, especially snakes, are persecuted, although only some of these are endangered species. This situation contrasts with that of amphibians, for which the authors state that direct human persecution is not significant and that the main threats to this group of wildlife are the destruction and alteration of habitat, pollution, and invasive species. Also, Cox and Temple [67], in a report by IUCN on the state of conservation of reptiles in Europe, reinforce the idea that the main threat to reptiles is the destruction and alteration of habitat, followed by over-exploitation, pollution, and direct persecution. As Brito et al. [36] state, there are several reports of snakes being deliberately killed throughout Europe in the nineteenth and early twentieth centuries and the extinction of some populations, especially poisonous snakes belonging to the genus *Vipera*. The Latastei viper (*Vipera latastei*) was driven to extinction in the Columbretes Islands during the construction of a lighthouse [69]. Also the adder (*Vipera aspis*) became extinct in the forests around Paris [70] and *Vipera ursinii rakosiensis *became extinct in Romania and Austria due to deliberate persecution and extermination [71]. In North America, rattlesnakes are persecuted through round-up events during which they are collected from their natural habitat and killed [72]. Whittaker and Shine [73] indicate that in Australia, in a study of the causes of mortality of big elapid snakes, 38% of the respondents to a questionnaire affirmed that they attacked snakes when they found them, for reasons of fear, hate, and concern for the safety of children or pets. In a study conducted in Canada using fake snakes and turtles placed in parts of the road not used by cars, Ashley et al. [74] observed that often these baits were run over in the parts of the road that are usually not driven on, suggesting that motorists intentionally deviated from the normal route to run over these animals, and snakes were run over more often than turtles.

The presence of many myths and folklore related to these animals presenting them as dangerous and venomous [24,35,36] may contribute to these misconceptions that people have about them. For further research, we aim to understand better how wrong perceptions can influence the presence of negative values and whether the clarification of these wrong perceptions can influence the manifestation of these types of values and change the persecutory attitudes toward these animals, allowing better plans to be made for their conservation. Also, given the failure of protective legislation to stop the capture and killing of these animals, we need an alternative approach. The obvious possibility is an increased emphasis on environmental education, as proposed by Whitaker and Shine [73]. Data from this study and from Ceríaco [35] suggest that such environmental education programs should focus on the clarification of the wrong perceptions about the degree of danger and usefulness of these animals and on better and clearer presentation of these animals' real characteristics (as opposed to folklore and aesthetic characteristics). Life history, ecological issues, and conservation problems should also be addressed, especially the potential usefulness of these animals as predators of pests and to the food-chain equilibrium. In order to protect animals which are part of a strong cultural heritage and regarding which a large number of stories and misconceptions exist, an interdisciplinary approach is essential. Such an approach includes the analysis of local folklore, as examination of misconceptions is necessary to understand not only why they still exist in the popular imagination, but also how they may constitute a real risk to the survival of the species in question.

Even if the animal related folklore, or if we prefer, ethnozoology should not be disregarded, and must even be considered as a very important socio-cultural heritage, sometimes it can clearly constitute a conservation problem. The case of geckos in southern Portugal [24] is a fine example of this, since we can consider the folklore that surround the geckos as a very old cultural heritage, that shall be studied, understood and preserved (and that even can constitute a very interesting stand point for history of culture and history of science studies), otherwise constitute the basis of why these animals are hated and persecuted, resulting in thousands of direct killings every year, and must be controlled and prevented through directed information and educational campaigns. Although, if the study of the complex socio-cultural context and the nature of these folklore, which is needed to better understand these human-animal relations, is very important, the present study serves to present a stand-point evidence that the presence of some wrong ideas, resulting from folklore, can clearly influence anti-conservationist and persecution attitudes towards amphibians and reptiles.

## Competing interests

The author declares that they have no competing interests.

## Authors' contributions

As single author LMPC was responsible for the design of the study, the data collection (where he was helped by some colleagues, referred in the Acknowledgments section.), data analysis and writing of the entire manuscript.
